# Predicting anemia using NIR spectrum of spent dialysis fluid in hemodialysis patients

**DOI:** 10.1038/s41598-021-88821-4

**Published:** 2021-05-18

**Authors:** Valentina Matović, Branislava Jeftić, Jasna Trbojević-Stanković, Lidija Matija

**Affiliations:** 1 Faculty of Mechanical Engineering, Belgrade University, Kraljice Marije 16, 11120 Belgrade, Serbia; 2 Faculty of Medicine, Belgrade University, Dr Subotica 8, 11000 Belgrade, Serbia; 3 Clinic of Urology, University Hospital Center “Dr Dragiša Mišović-Dedinje”, Heroja Milana Tepića 1, 11000 Belgrade, Serbia

**Keywords:** Computational biology and bioinformatics, Nephrology

## Abstract

Anemia is commonly present in hemodialysis (HD) patients and significantly affects their survival and quality of life. NIR spectroscopy and machine learning were used as a method to detect anemia in hemodialysis patients. The aim of this investigation has been to evaluate the near-infrared spectroscopy (NIRS) as a method for non-invasive on-line detection of anemia parameters from HD effluent by assessing the correlation between the spectrum of spent dialysate in the wavelength range of 700–1700 nm and the levels of hemoglobin (Hb), red blood cells (RBC), hematocrit (Hct), iron (Fe), total iron binding capacity (TIBC), ferritin (FER), mean corpuscular volume (MCV) and mean corpuscular hemoglobin concentration (MCHC) in patient blood. The obtained correlation coefficient (R) for RBC was 0.93, for Hb 0.92, for Fe 0.94, for TIBC 0.96, for FER 0.91, for Hct 0.94, for MCV 0.92, for MCHC 0.92 and for MCH 0.93. The observed high correlations between the NIR spectrum of the dialysate fluid and the levels of the studied variables support the use of NIRS as a promising method for on-line monitoring of anemia and iron saturation parameters in HD patients.

## Introduction

Chronic kidney disease (CKD) is a highly prevalent and ubiquitous disease affecting between 4 and 14% of adults worldwide. It is estimated that 5 million individuals will require renal replacement therapy for end-stage renal disease by the year 2030, presenting a substantial burden on health services worldwide^1,2^. Anemia is a well-known consequence of CKD, and is associated with structural and functional alterations of myocardium in this population, thus contributing to the risk of cardiovascular morbidity and mortality^3^. The kidney is the major source of erythropoietin, and the ability to secrete this hormone is lost as the kidney function declines. Other factors contributing to anemia include impaired response of the bone marrow to erythropoietin caused by uremic milieu and chronic inflammation, absolute and relative iron deficiency, a shortened erythrocytes' half-life, vitamin deficiencies precipitated by malnutrition and diet restrictions, and blood losses related to hemodialysis (HD).

Anemia is defined as a red blood cell (RBC) count that is not sufficient for delivering oxygen to peripheral tissues^4^. The hemoglobin (Hb), hematocrit (Hct) and/or the RBC concentrations can be used to establish the presence of anemia in a patient^4^. On the other hand, the qualitative characteristics of the red cell population are defined by the mean corpuscular volume (MCV) and the mean corpuscular hemoglobin concentration (MCHC)^4^. Iron deficiency is the most common nutritional deficiency in the world^5^. The RBC and serum ferritin (FER) concentrations, along with the transferrin saturation can detect earlier changes in iron status^6^. The ferritin is an indicator of the total body iron stores, while the total iron binding capacity (TIBC) can be used as an estimator of serum transferrin^7^.

The current recommendations for anemia monitoring in HD patients require hemoglobin monitoring every 2 to 4 weeks, thus necessitating frequent blood sampling, which further contributes to anemia^8^. On the other hand, studies have shown that clinically optimal Hb monitoring would actually be on a weekly basis because short-term Hb variability affects the reliability of Hb measurement and may lead to incorrect dosing of erythropoiesis stimulating agents^9^. Thus, for the HD population, an on-line monitoring method of anemia and iron levels might be clinically valuable and patient-friendly as it would avoid frequent blood sampling, but still provide important information on the patients' status.

## Background

The possibility of detecting certain substances (urea, creatinine) in the spent dialysate fluid has already been proven by Fridolin et al.^10,11^ in UV region. Henn et al. showed the possibility to detect biological substances (urea, glucose, lactate, phosphate and creatinine) in the MIR region using artificial dialysate fluid^12^. On-line scanning of blood and monitoring of substituents is complicated by the fact that blood is a highly saturated fluid, prone to coagulation and clotting. All subcutaneous biosensors suffer from interferences in complex matrices such as blood or serum. Coating of the sensor with proteins or cellular material from the biological matrix is also a frequent phenomenon^13^. Optical sensors that receive an array of light emitted through a spent dialysis fluid that contains blood substituents and use the principle of NIR absorption pattern may overcome these interferences. This signal can then be quantitatively related to the blood substituents concentration^14^. Such approach is nondestructive and information about multiple analytes can be obtained from a single NIR spectrum^15,16^, representing a great potential for measurement and detection of blood components^17,18^.

To the best of our knowledge, there is no previous work on automatic anomaly detection of blood indices levels based on the scanning of the spent dialysate. There have been relevant studies in the area of spent dialysate monitoring, but the specific problem of monitoring and detecting anomalies in blood indices levels using the NIR spectrum has not been addressed. Moreover, a machine learning (ML) approach has not been adopted for this purpose^10,19^.

In this study we demonstrate the utility of NIR analysis for the indirect measurement of anemia related parameters: RBC, Hb, Hct, MCV, MCH, MCHC, Fe, FER and TIBC. The objective was to evaluate the accuracy of NIR spectroscopy of the spent dialysate fluid for determining concentrations of these analytes by assessing the correlation between the spectra and values of Hb, RBC, Hct, MCV, MCH, MCHC, Fe, TIBC and FER in the blood circulation.

## Materials

### Subjects and dialysis parameters

Samples of spent dialysis fluid were collected from 35 maintenance HD patients, of whom 9 were treated with hemodiafiltration (HDF) and 26 with high-flux HD. The samples have been collected during the course of a year. Inclusion criteria were stable HD prescription, stable intradialytic blood pressure, the absence of physical weakness or dyspnea, and the ability to rest in a 45°–90° position during the entire dialysis session. The dialysis was performed using Dialog + Adimea, (BBraunAvitum AG, 34209 Melsungen, Germany) machines. The dialysate contained Na+ 138 mmol/l, Cl 110.5 mmol/l, K+ 2 mmol/l, Ca++ 1.75 mmol/l or 1.50 mmol/l, Mg++ 1 mmol/l, CH3COO 3 mmol/l, HCO3 32 mmol/l, Glucose 1 g/l. The mean dialysate flow was 500 ml/min, and the mean effective blood flow was 300 ml/min. All patients were dialyzed via antebrachial arterio-venous fistulas using a two-needle system.

### Sampling procedures

The samples of spent dialysate were collected directly from the dialyzer outlet during the dialysis session. It was previously ensured that the dialysate flow was free and uninterrupted. The spent dialysate, containing dialyzed waste metabolites, flowed upwards through the cartridge, and the outlet to the external environment. Fifteen milliliters of spent dialysate fluid were sampled from the effluent line 15 min after the beginning of the dialysis session. Visible-Near infrared (VIS–NIR) absorbance spectra of the samples were measured on the following day.

The blood samples were collected from the sampling port on the arterial line of the dialysis system, e.g., coming from the patient immediately before entering the dialysis circuit. Composition of biological analytes used in this study are shown in Table 1.

**Table 1:** Composition of biological analytes used in this study.

Parameter	Mean ± SD	Max value	Min value	Max predicted value	Min predicted value
RBC (10^12^ l )	3.49 ± 0.309	4.17	2.68	4.2	2.77
Hb (g/l)	105.57 ± 7.82	82	119	122	83
Hct (l/l )	0.34 ± 0.025	0.39	0.267	0.388	0.266
Fe (mcmol/L)	11.33 ± 2.99	19	8	19.27	6.86
TIBC (%)	42 ± 7.52	54	28	52.6	26.4
FER (ng/ml)	377 ± 76.97	471	225	480	246
MCV (fL)	97.446 ± 6.46	104.1	88.8	103.7	89
MCHC (g/dL)	310 ± 7.54	326	297	332	297.2
MCH (pg)	30.7 ± 1.318	27.5	32.4	32.6	26,95

## Methods

Ultraviolet–Visible-Near infrared (UV–VIS–NIR) optical absorption spectra of the spent dialysate have been registered using the spectrometer Lambda 950 (PerkinElmer CA, USA). The wavelength region of interest was 700–1700 nm, and the resolution was set to 2 nm. Absorbance spectra were scanned and collected three times per sample. The optical path-length was 1 mm.

### Blood parameter analysis

Complete blood count and all related variables were determined by fluorescent flow cytometry on the Sysmex XS-1000i hematological analyzer (Sysmex Corporation, Japan). Hemoglobine was determined by sodium lauryl sulphate method. Serum iron and ferritin levels were measured with the Dimension RxL Max analyzer (Siemens Healthcare GmbH, Germany).

The ethical committee of the University Hospital Center Dr Dragiša Mišović has reviewed the study protocols and signed informed consent forms have been obtained from all participating patients. All the methods described herein have been performed in accordance with the relevant guidelines and regulations.

### Preprocessing

Mathematical pre-processing such as scatter correction and derivatives has been investigated and the best one was selected on the basis of the lowest standard error and highest coefficient of determination. The best results have been obtained with the Standard Normal Variate (SNV) technique.

### Artificial neural network algorithm

NIR absorption values were implemented into the ANN (artificial neural network) algorithm. Artificial neural networks (ANN) were originally designed to mimic the function of the human brain. They consist of a number of simple processing units (or neurons) linked by weighted modifiable interconnections. ANN is a flexible modeling methodology, since both linear and non-linear functions can be used in the processing units. The backpropagation ANN is the most common neural network. It generally consists of an input layer, one or more hidden intermediate layers and one output layer. A neuron is a processing unit that transforms, by an activation function, an input into output data. Once the ANN model is trained, the analysis of its connection weights can easily identify the important inputs^20^.

Here, in order to form the ANN and perform its training, the MATLAB Neural Net Fitting tool (nftool) was used. The NIR spectrum of the spent dialysis fluid is used as input, while the red blood parameters were taken as output. The data are randomly divided into two groups: 85% of data is used for training and the remaining 15% is used for testing.

There are several batch training algorithms that can be used to train a network, like Levenberg–Marquardt and Scaled Conjugate Gradient. In this research, the Bayesian regularization training algorithm has been used. This function updates the values of weights and biases according to the Bayesian optimization method so as to minimize the network error.

The test set data have no effect on the training process. This set provides an independent measure of network performance during and after training. The training starts with 2 and finishes with 10 hidden neurons. The number of hidden layer neurons is increased when the network is not performing well. The optimum number of hidden neurons was determined to be three. Training multiple times generates different results due to different initialization of connection weights and different initial conditions.

There are a number of solutions based on ML that provide support to physicians and medical professionals. ML provides advantages for recognizing complex correlative relations between the input variables. By reducing the utilization of redundant information in input variables in the course of the training process, the ML algorithms produce highly nonlinear decision boundaries, permitting the use of small training data samples, and simply exploit various forms of medical data that may be latent in nature. Machine Learning has been recognized as a helpful tool for decision making in both diagnosis and medical treatment. Several applications of ML in medicine have been reported to produce an excellent fit of the model to a given set of data^21,22^.

### Ethics approval

The ethical committee of the University Hospital Center Dr Dragiša Mišović has reviewed and approved the study protocols and signed informed consent forms have been obtained from all participating patients.

### Consent to participate

Written informed consent was obtained from the patients.

### Consent for publication

The author hereby consents to publication of the work.

## Results

The following regression plots display the network outputs with respect to targets for training and test sets (Figs. 1, 2, 3, 4, 5, 6, 7, 8, 9). For a perfect fit, the data should fall along the 45-degree dashed line, where the network outputs are equal to the targets.

**Figure 1:**
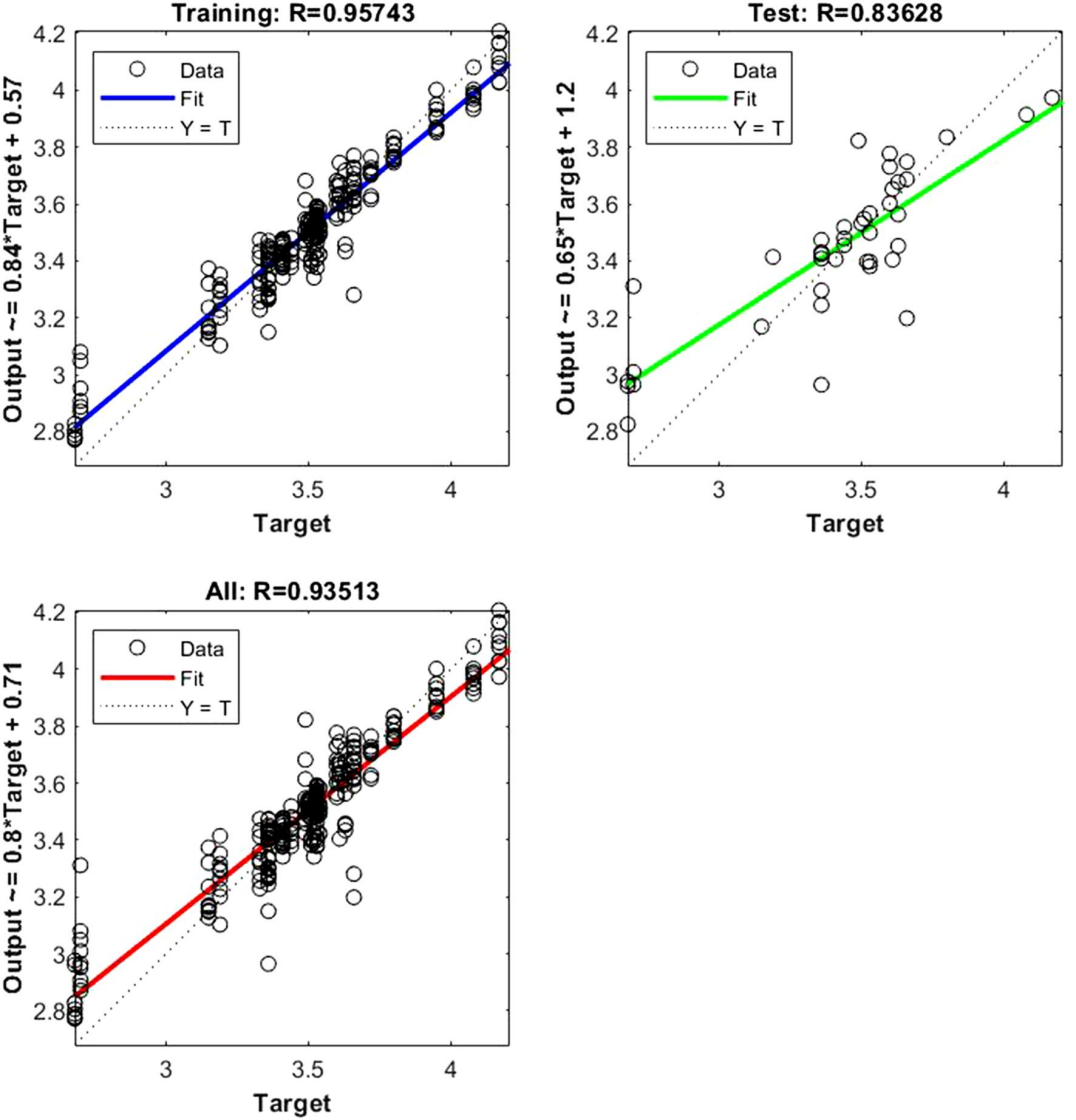
Shows the regression plot between the NIR-absorbance of spent dialysate and the RBC concentration in the patient blood at the beginning of the HD session (wavelength from 700–1700 nm, R_all_ = 0.93, R_train_ = 0.95, R_test_ = 0.83, the number of spectra used for training was N = 270). The RBC concentration in patients’ blood was 3.49 ± 0.309·10^12^ l (mean ± SD). The R_all_ represents the value of R for the training and test sets. The equation relating the predicted and measured values is Output = 0.8*Target + 0.71.

**Figure 2:**
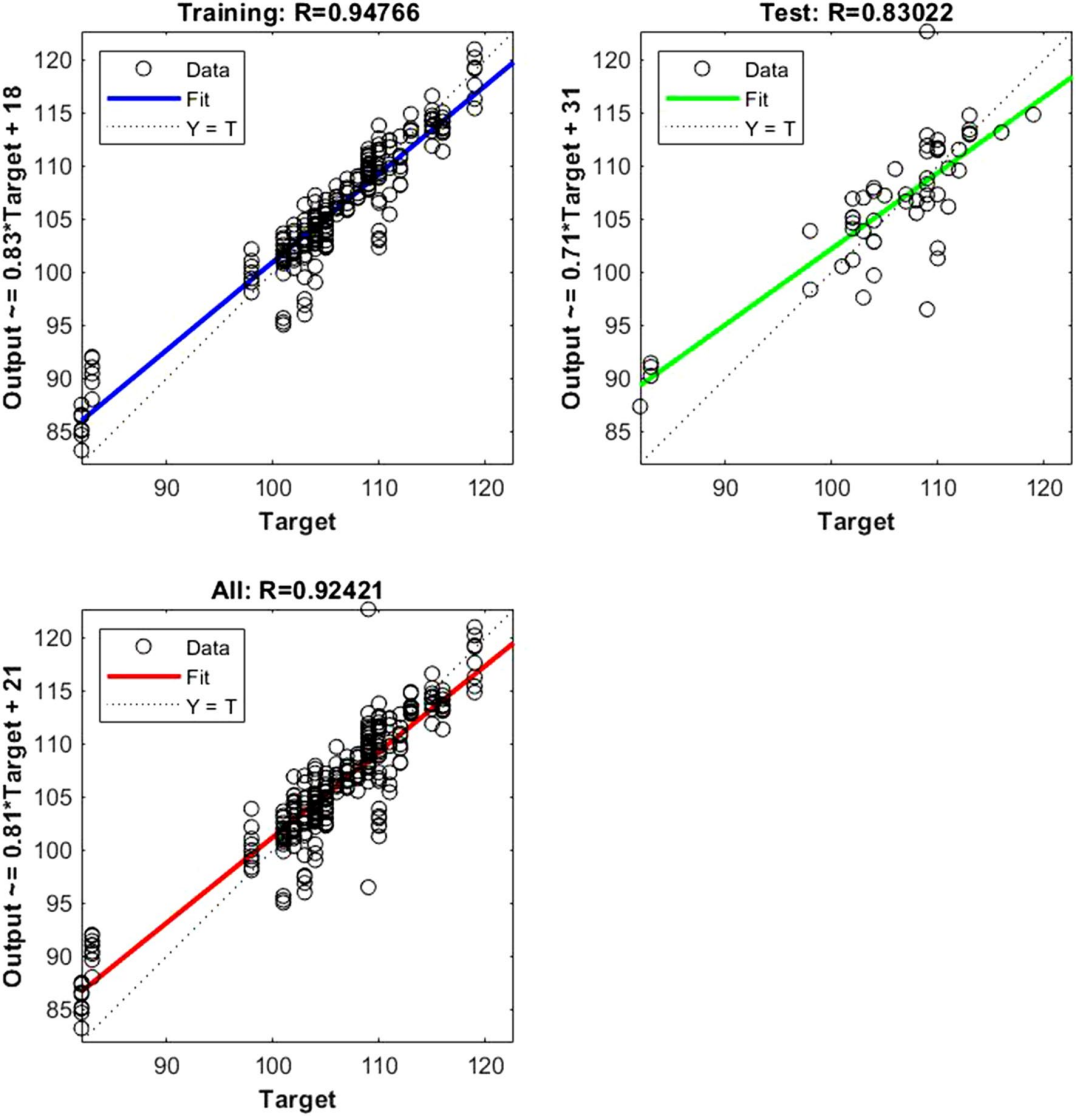
Shows the regression plot between the NIR-absorbance of spent dialysate and the Hb concentration in the patient blood at the beginning of the HD session (wavelength from 700–1700 nm, R_all_ = 0.92, R_train_ = 0.94, R_test_ = 0.83, number of spectra used for training was N = 270). The Hb concentration in patients' blood was 105.57 ± 7.82 g/l (mean ± SD). The equation relating the predicted and measured values is Output = 0.81*Target + 21.

**Figure 3:**
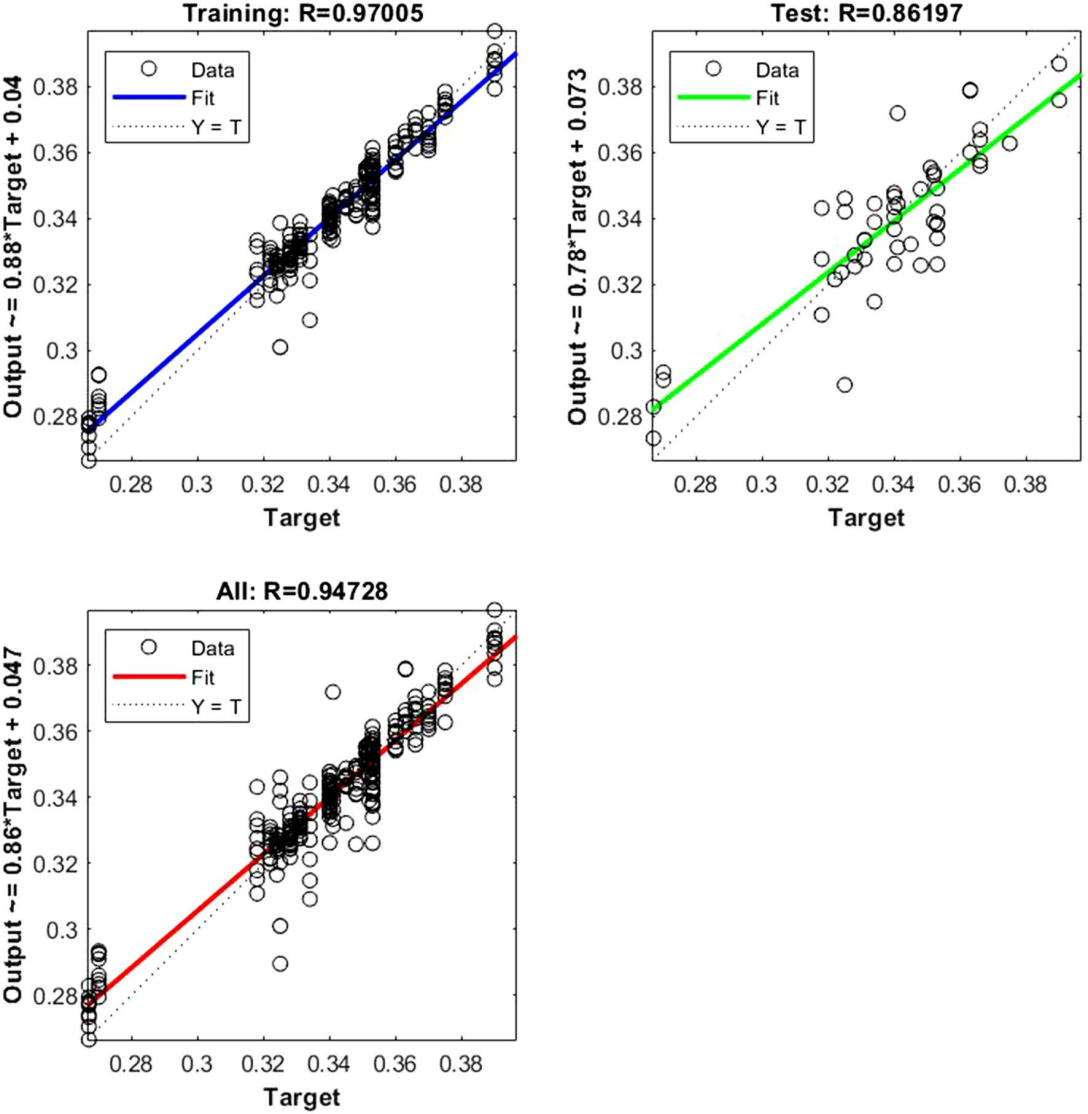
Shows the regression plot between the NIR-absorbance of spent dialysate and the hematocrit concentration in the patient blood at the beginning of the hemodialysis session (wavelength from 700–1700 nm, R_all_ = 0.94, R_train_ = 0.97, R_test_ = 0.86, number of spectra used for training was N = 270). The hematocrit concentration in patients’ blood was 0.34 ± 0.025 l/l (mean ± SD). The equation relating the predicted and measured values is Output = 0.86*Target + 0.047.

**Figure 4:**
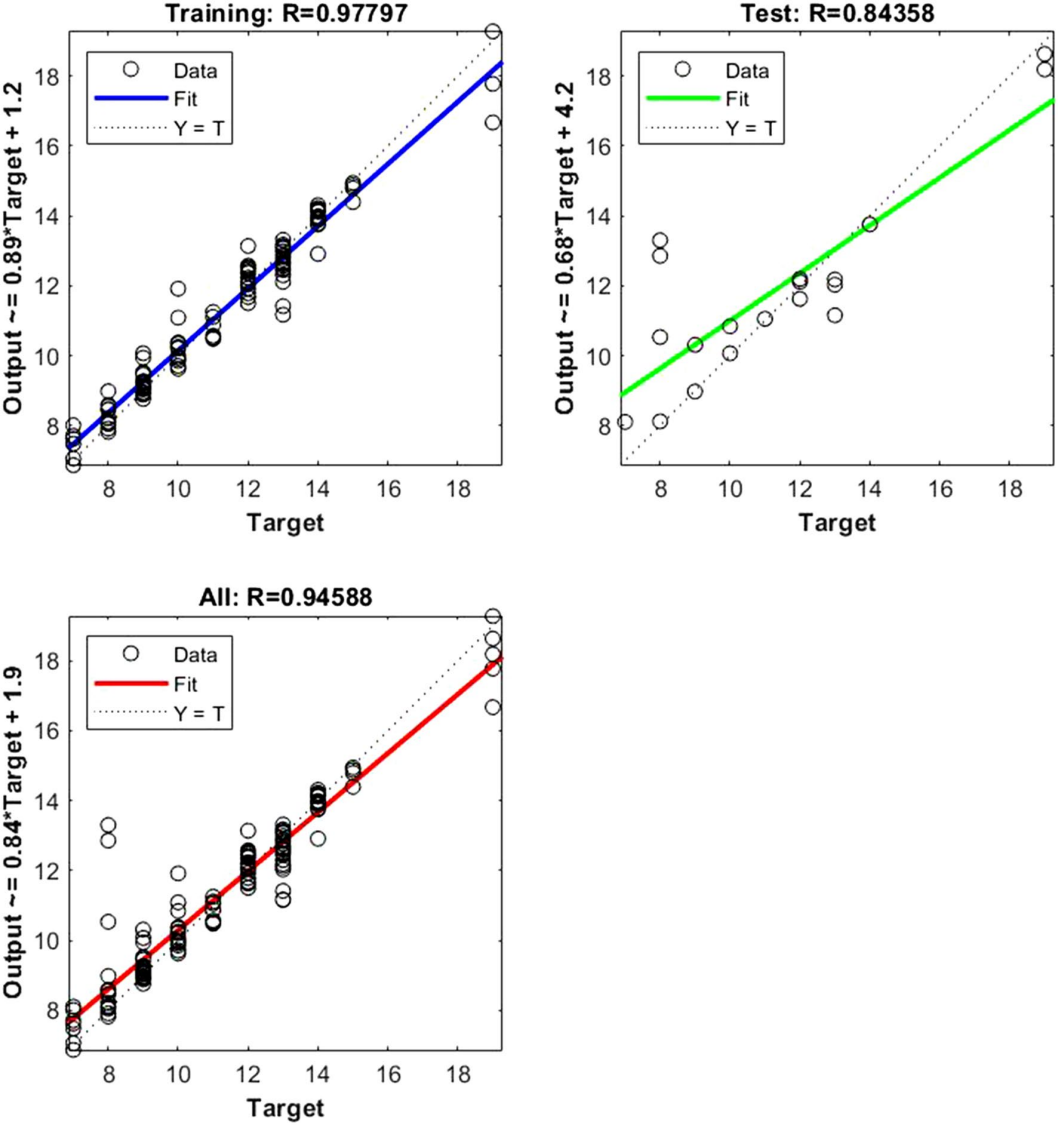
Shows the regression plot between the NIR-absorbance of spent dialysate and the Fe concentration in the patient blood at the beginning of the hemodialysis session (wavelength from 700–1700 nm, R_all_ = 0.94, R_train_ = 0.97, R_test_ = 0.84, number of spectra used for training was N = 126). The Fe concentration in patients’ blood was 11.33 ± 2.99 (mean ± SD). The TIBC levels were 42 ± 7.52%, and the FER 377 ± 76.97 ng/ml. The equation relating the predicted and measured values is Output = 0.84*Target + 1.9.

**Figure 5:**
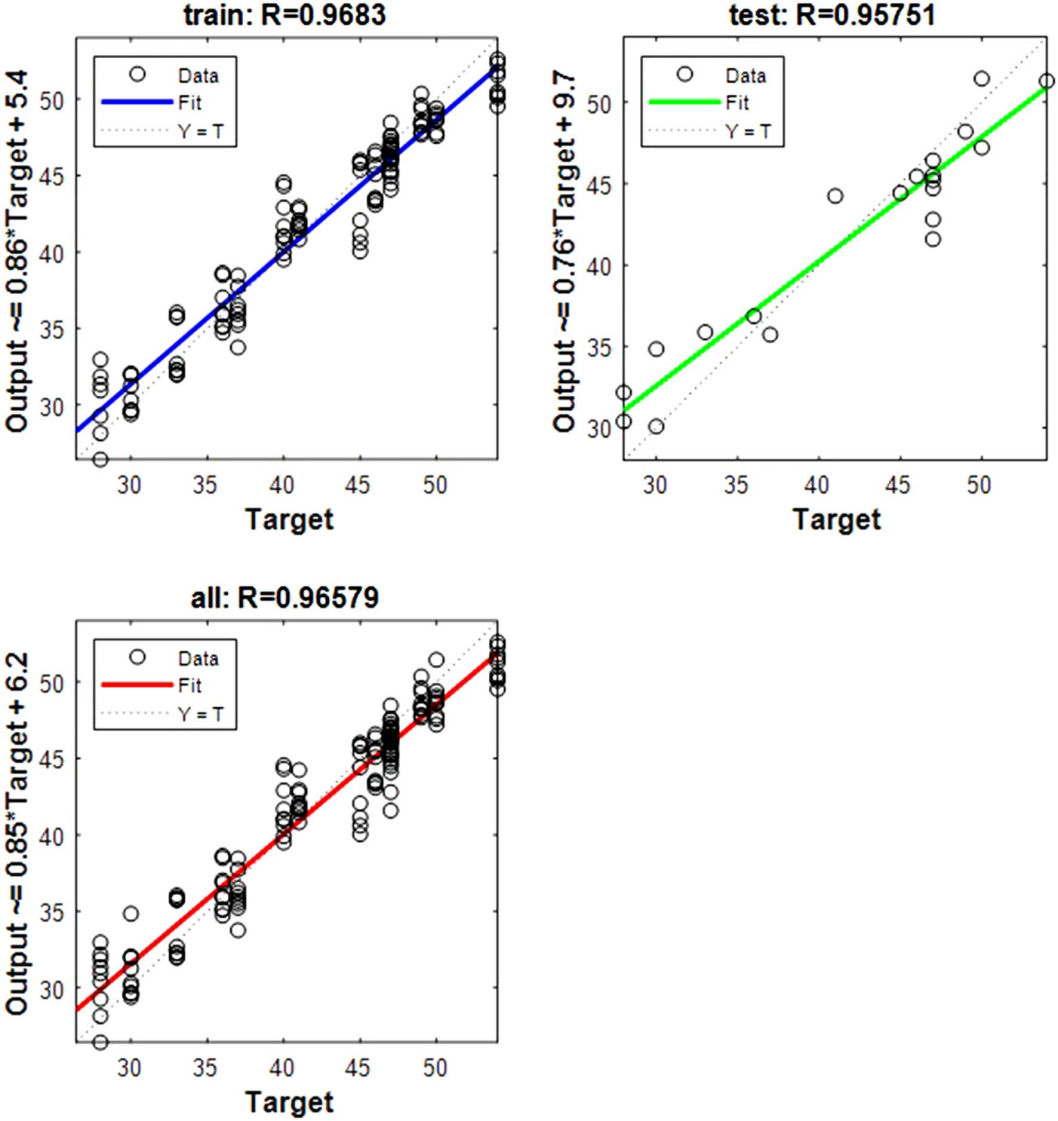
Shows the regression plot between the NIR-absorbance of spent dialysate and the TIBC value in the patient blood at the beginning of the hemodialysis session (wavelength from 700–1700 nm, R_all_ = 0.96, R_train_ = 0.96, R_test_ = 0.95 number of spectra used for training was N = 135). The equation relating the predicted and measured values is Output = 0.85*Target + 6.2.

**Figure 6:**
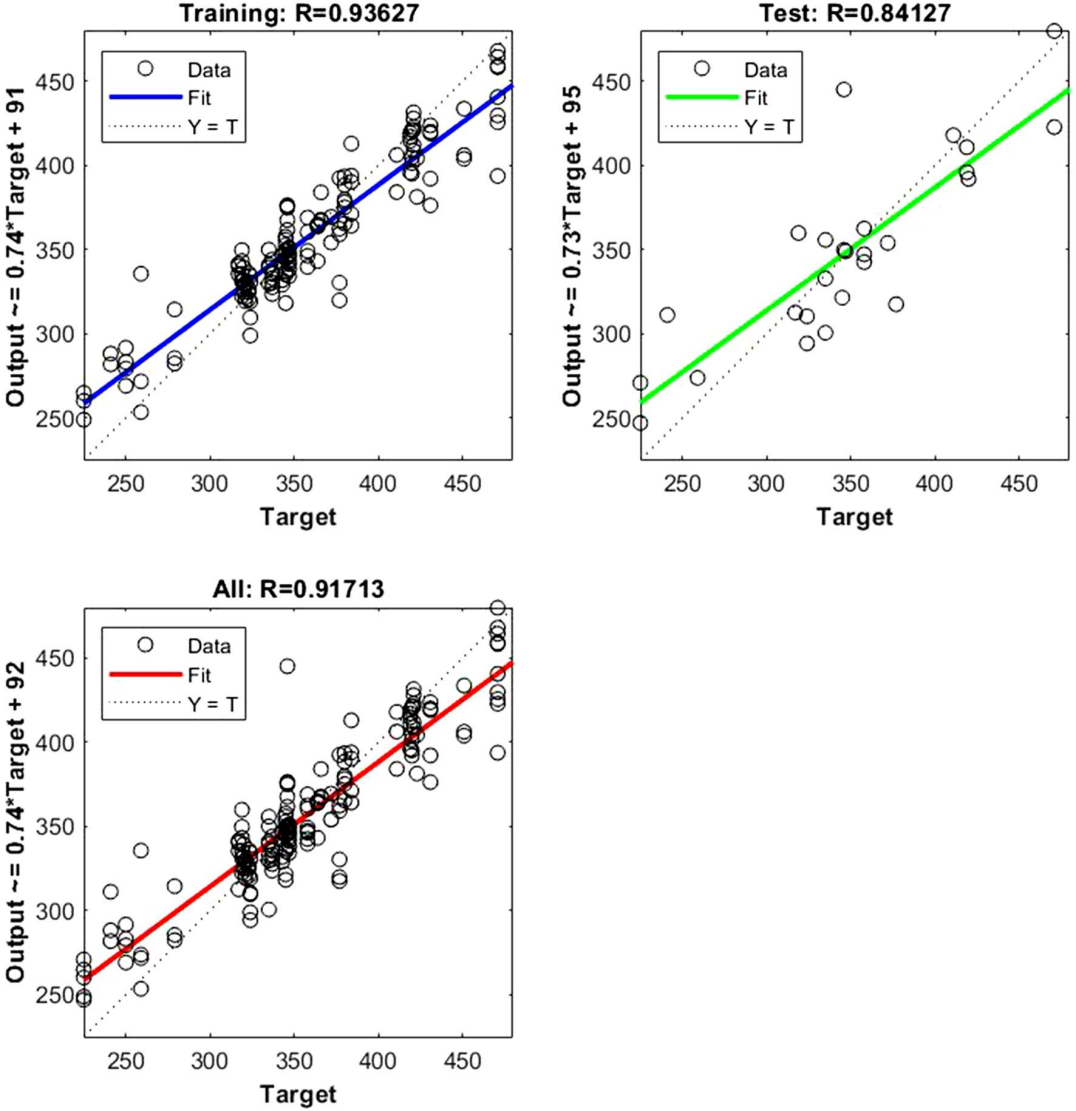
Represents the regression plot between the NIR-absorbance of spent dialysate and the FER concentration in the patient blood at the beginning of the hemodialysis session (wavelength from 700–1700 nm, Rall = 0.91, R_train_ = 0.93, R_test_ = 0.84, number of spectra used for training was N = 108). The equation relating the predicted and measured values is Output = 0.74*Target + 92.

**Figure 7:**
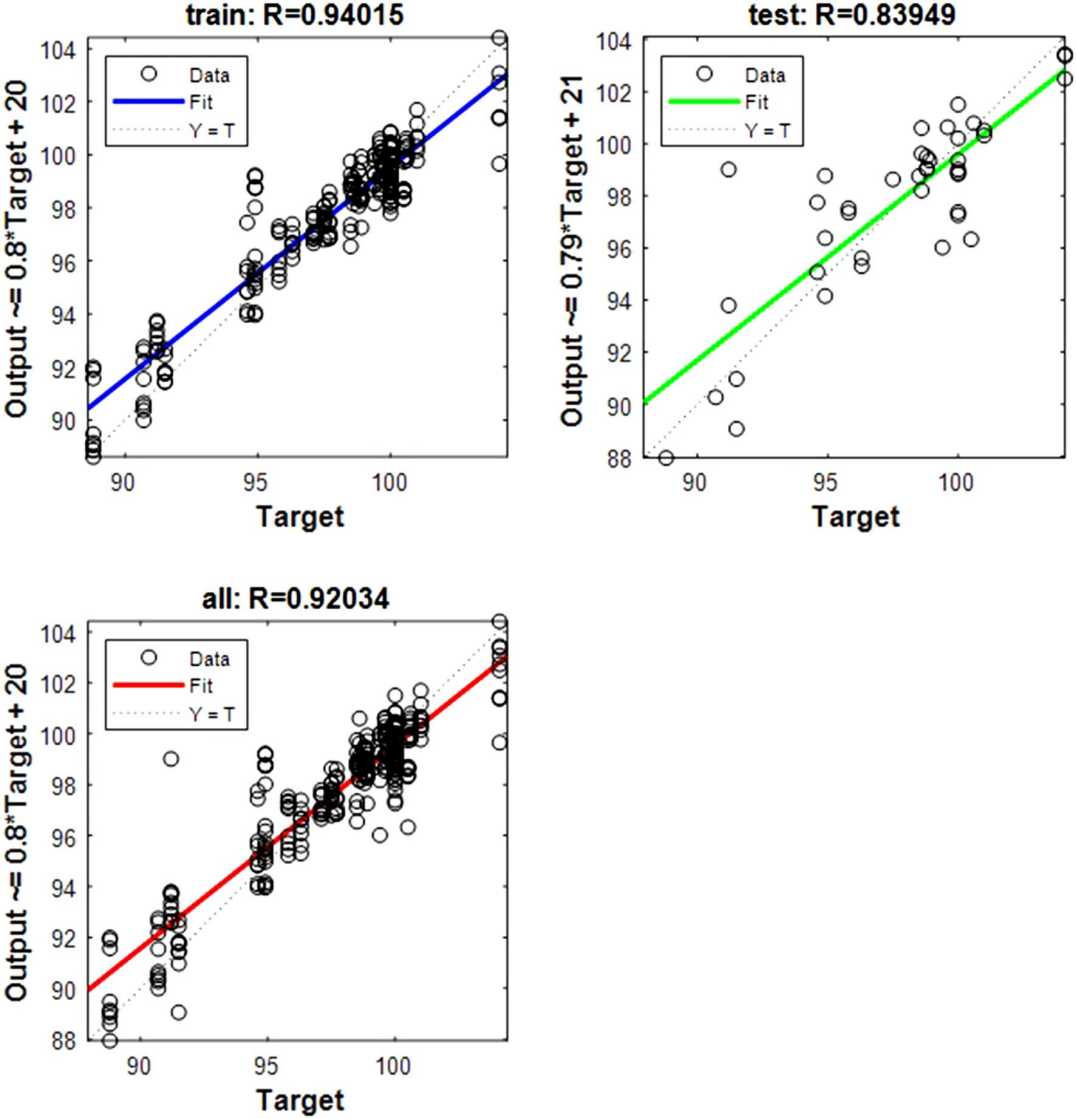
Shows the regression plot between the NIR-absorbance of spent dialysate and the MCV concentration in the patient blood at the beginning of the hemodialysis session (wavelength from 700–1700 nm, R_all_ = 0.92, R_train_ = 0.94, R_test_ = 0.83, number of spectra used for training was N = 270). The MCV concentration in patients’ blood was 97.446 ± 6.46 (mean ± SD). The equation relating the predicted and measured values is Output = 0.8*Target + 20.

**Figure 8:**
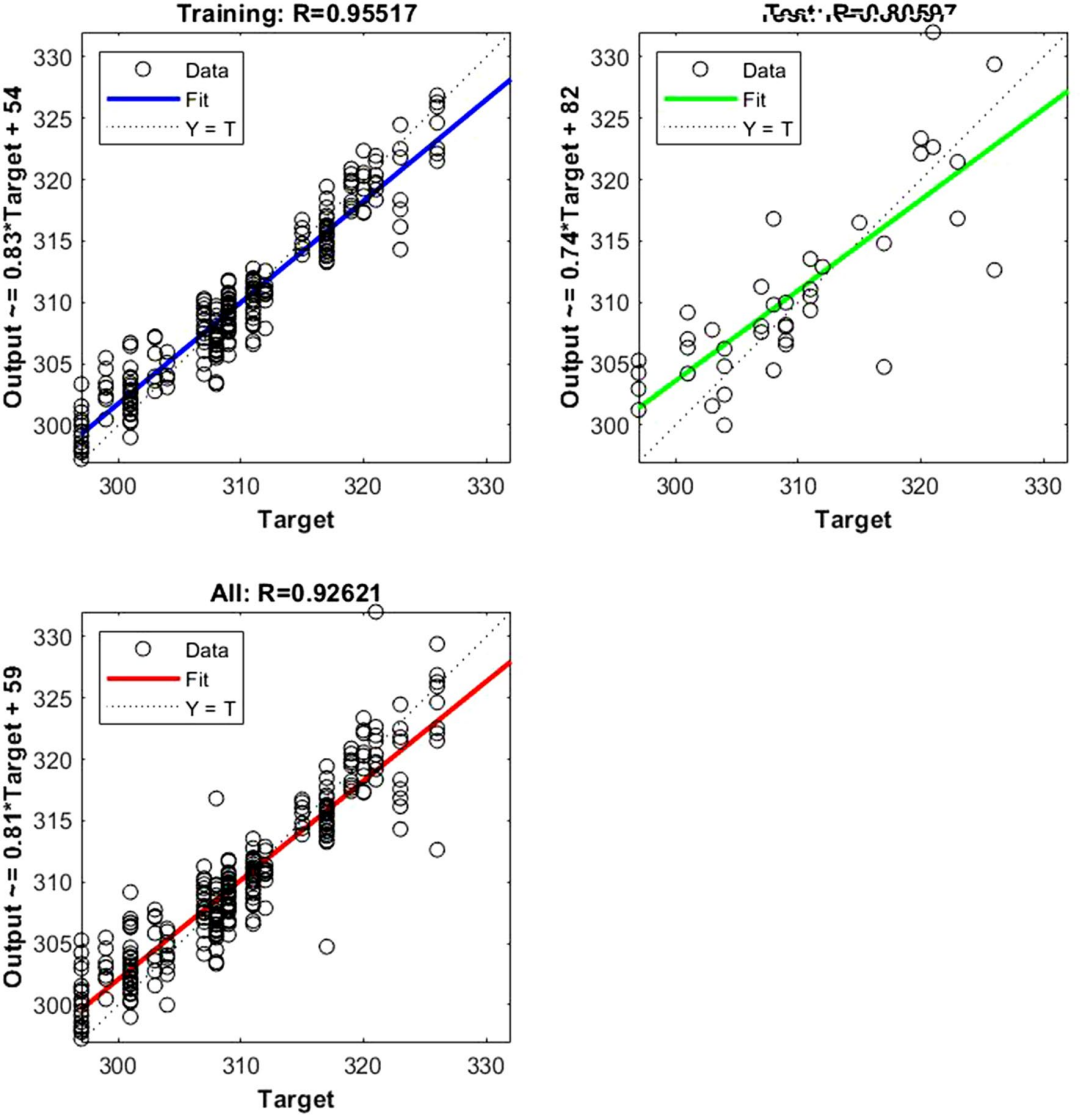
Represents the regression plot between the NIR-absorbance of spent dialysate and the MCHC concentration in the patient blood at the beginning of the hemodialysis session (wavelength from 700–1700 nm, R_all_ = 0.92, R_train_ = 0.95, R_test_ = 0.80, number of spectra used for training was N = 270). The MCHC concentration in patients' blood was 310 ± 7.54 (mean ± SD). The equation relating the predicted and measured values is Output = 0.81*Target + 59.

**Figure 9:**
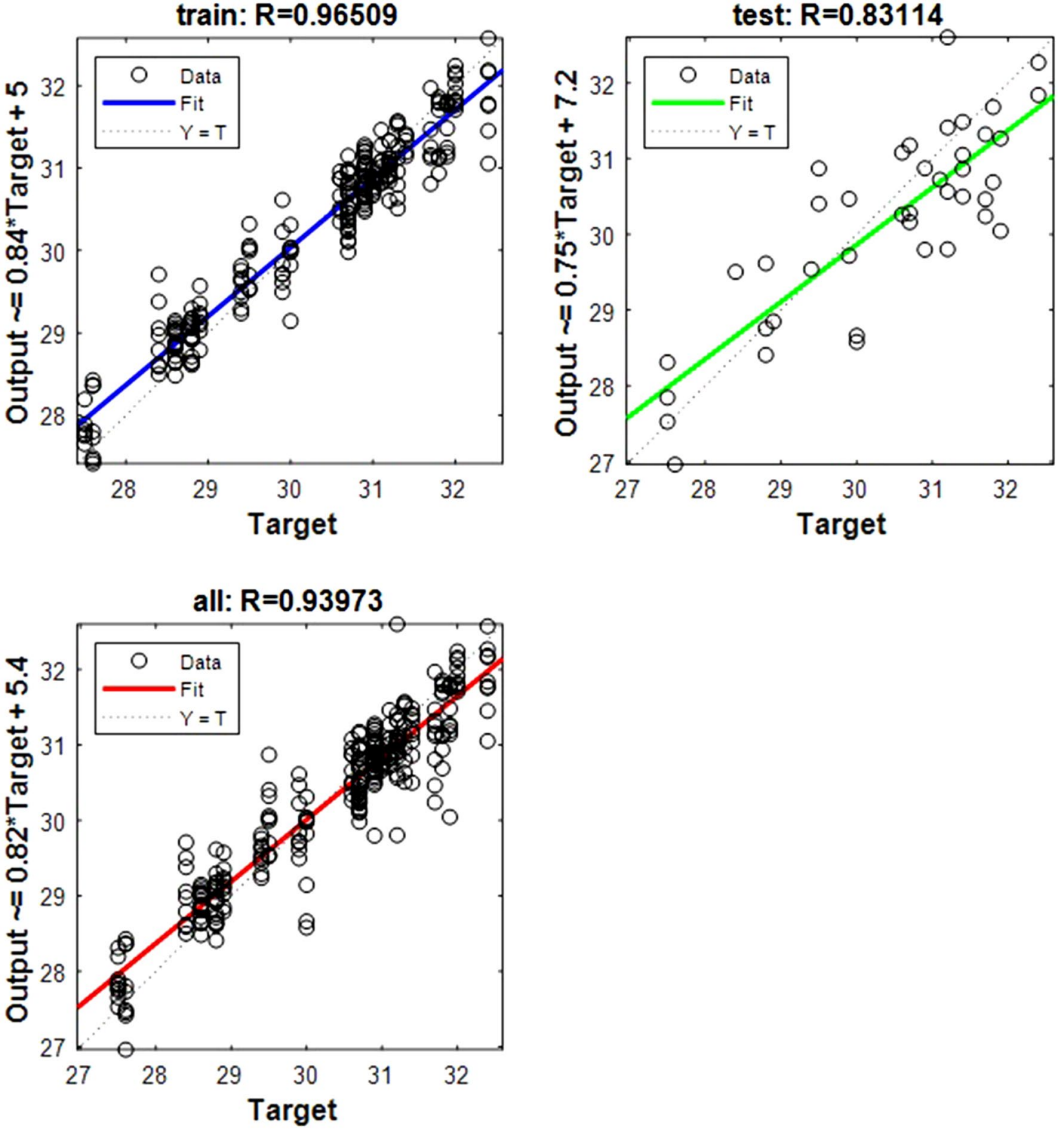
Represents the regression plot between the NIR-absorbance of spent dialysate and the MCH concentration in the patient blood at the beginning of the hemodialysis session (wavelength from 700–1700 nm, R_all_ = 0.93, R_train_ = 0.96, R_test_ = 0.80, number of spectra used for training was N = 270). The MCH concentration in patients' blood was 310 ± 7.54 (mean ± SD). The equation relating the predicted and measured values is Output = 0.82*Target + 5.4. Absorption spectra of the spent dialysate fluid for different values of hemoglobin concentration are shown in Fig. 10.

**Table 2:** Statistical parameters of the machine learning algorithm.

	RBC (10^12^ l)	Fe (mcmol/L)	FER (ng/ml)	Hct (l/l)	Hb (g/l)	MCH (pg)	MCHC (g/dL)	MCV (fL)	TIBC (%)
R	0.935	0.945	0.917	0.947	0.924	0.939	0.926	0.92	0.965
p-value:	2.20E16	2.20E16	2.00E16	2.20E16	2.20E16	2.20E16	2.20E16	2.20E16	2.20E16
R-squared:	0.874473	0.894688	0.841133	0.8923	0.854169	0.88309	0.8579	0.847018	0.932755
sse	22	842	479,002	0.1436	14,057	423	13,331	2743	7085
SSR	3.231768	99.17393	90,470.25	0.017341	2400.08	56.13242	2208.897	495.5051	510.7922
RPD	2.753501	3.037185	2.426428	3.033819	2.598154	2.864682	2.627651	2.536246	3.629581
RMSEP	0.112354	0.903719	23.57733	0.008283	3.010562	0.466367	2.892583	1.36816	2.074357
MSE	0.012623	0.816708	555.8905	6.86E05	9.063482	0.217498	8.367035	1.871861	4.302958
Bias	− 0.00436	− 0.08932	− 0.206004	− 3.51E06	− 0.11802	0.040105	− 0.16751	− 0.007	0.26631
SEP	0.1122694	0.89929415	23.57643	0.008283	3.0082478	0.4646394	2.8877287	1.3681421	2.0571913
Max value	4.17	19	471	0.39	119	32.4	326	104.1	54
Min value	2.68	8	225	0.267	82	27.5	297	88.8	28
RER	13.271652	12.23181536	10.43415	14.849693	12.299519	10.545813	10.042495	11.183049	12.638591

The data points are represented with circles. The R value is an indication of the relationship between the neural network outputs and targets. The correlation coefficient (R-value) measures the correlation between outputs and targets. Correlation was considered perfect if *R* was = 1 (there is an exact linear relationship between outputs and targets), very strong if *R* was > 0.90 and < 1, moderate if *R* was > 0.60 and < 0.80, and fair if *R* was < 0.60^23^. If R is close to zero, no linear relationship between outputs and targets exists.

The following regression plots display the neural network outputs with respect to targets for training and test sets.

The MSE for training and testing sets, quantifying the difference between the network outputs and measured values, are very close to zero. Therefore, it can be assumed that the designed ANN model is well trained by observing the R an MSE values.

**Figure 10:**
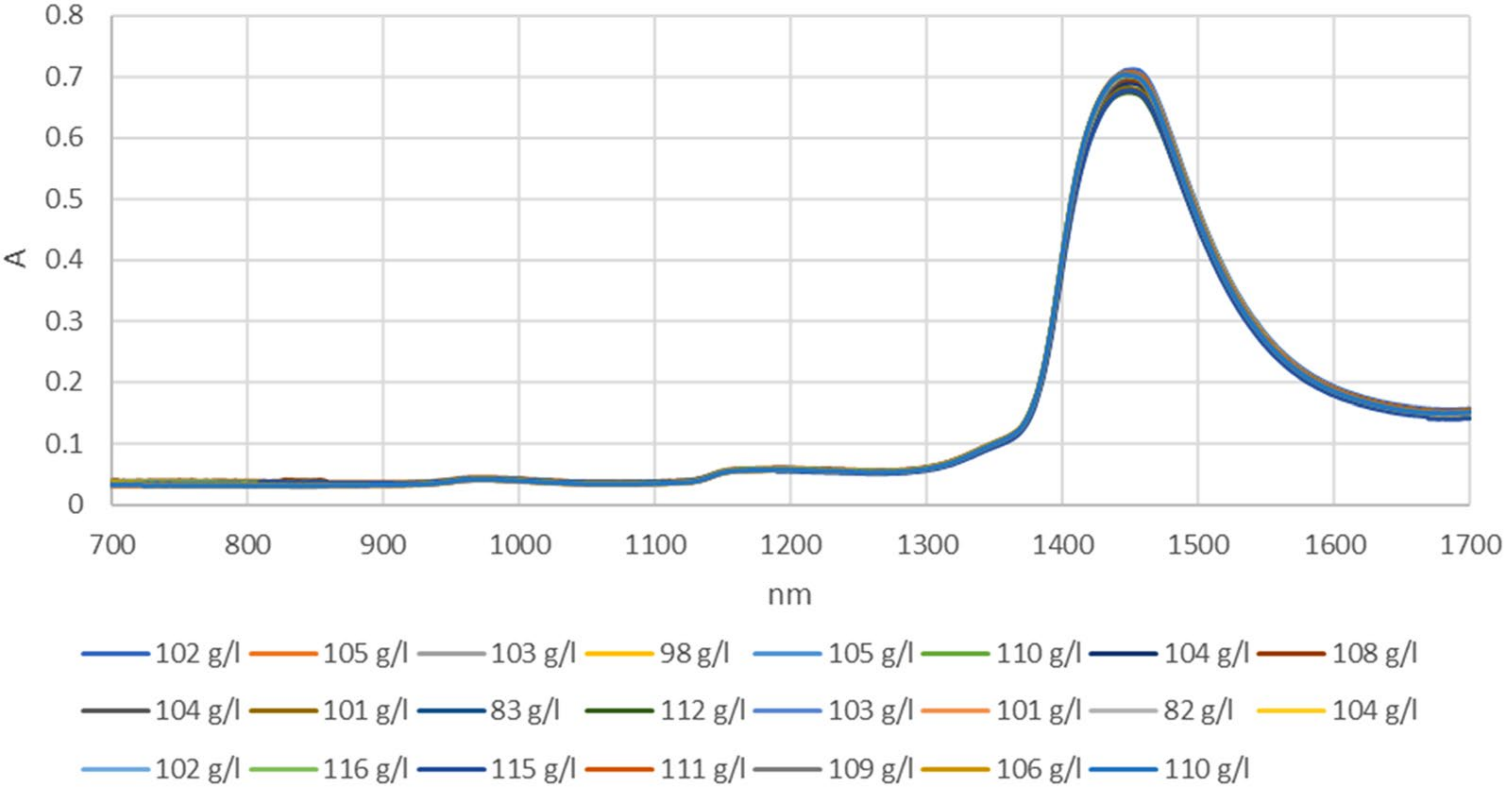
The absorption spectrum of the spent dialysate fluid shown as a function of hemoglobin concentration. R values for training and testing datasets are given in Table 3. The R values are very close to one another for both training and testing datasets. This means that the correlation between the network and the intended outputs does not stem from coincidence.

The best models are defined as those that yield the lowest values of the root mean square error in cross validation (RMSE), and the highest coefficient of determination. Furthermore, we have calculated the RPD (Table 2). Regarding the RPD statistic, an RPD < 2 is considered insufficient for applications, whereas a value for RPD between 2 and 2.5 makes approximate quantitative predictions possible. For values between 2.5 and 3, predictions can be classified as good, and an RPD > 3 indicates an excellent prediction^24^ (Fig. 10).

**Table 3:** R value for training and testing.

Parameter	R train	R test	R all
RBC	0.957	0.836	0.935
Fe	0.977	0.843	0.945
Fer	0.936	0.841	0.917
Hct	0.97	0.861	0.947
Hb	0.947	0.83	0.924
MCH	0.965	0.831	0.939
MCHC	0.955	0.805	0.926
MCV	0.94	0.839	0.92
TIBC	0.968	0.957	0.965

## Discussion

NIR spectroscopy is used as an alternative, non-invasive method for clinical analyses. In this method, NIR light is transmitted through or absorbed by the sample, and the substance concentration is predicted by analyzing the spectral information. Even information about complex substances can be obtained from a single NIR spectrum^25^. The goal of this study was to estimate the analytical benefit of NIR spectroscopy for the detection of anemia and iron indices from spent dialysate fluid in patients on maintenance HD.

The NIR region of the electromagnetic spectrum covers the wavelength range of 750–2500 nm^26^. We have empirically determined the optimal wavelength segment to be between 700 to 1700 nm.

In order to be used in clinical measurements, quantitative methods must be accurate and precise, reliable and inexpensive. The measurement procedures should be readily automated and the results should be quickly available. The NIR spectroscopy has the potential to satisfy all these conditions. Reagents are not required, nor sample preparation. The method is rapid and nondestructive, and is suitable for complex matrices. This makes it suitable for conducting low cost repetitive analyses.

The overtone and combination bands of the molecular vibrations of C–H, S–H, O–H and N–H bonds present in biological materials result in the appearance of near-infrared absorption bands that are wide and weak. When several biomolecules with similar concentrations are present in a matrix, the absorption bands overlap one another, so that at any given wavelength, many substances may contribute to the resulting spectrum^27^.

Blood Hct is routinely determined by analysis of blood samples. Friebel et al. introduced a non-invasive method for measuring Hct using the NIR spectroscopy (250 nm and 1100 nm)^34^. The occurrence of the NIR Fe absorption bands in the 700–1000 nm region is explained by the electronic transitions between the central iron ion and porphyrin ring and the transition between the ion and the coordinating oxygen^35^. The MCV, MCH, and MCHC were first introduced by Wintrobe in 1929 to define the size (MCV) and hemoglobin content (MCH, MCHC) of red blood cells^36^.

Blood parameters during hemodialysis (HD) are typically estimated from non-invasive measurements of their optical, electrical, acoustic or viscous properties while flowing through the dialyzer lines^37,38^. The classical way to measure red blood cell volume is by dilution technique, using 51Cr-labelled red blood cells, but this method is inpractical and seldom used in routine clinical practice.

Considering the rheologic properties of blood and its typically non-Newtonian behavior, some hypotheses can be formulated. Blood is a fluidized suspension of red blood cells with viscoelastic properties that reflect the cumulative effects of plasma viscosity and Hct. Based on the knowledge that the dialysis fluid has a constant chemical composition prior to contact with the dialyzer membrane, we can learn a lot about blood components based on substances that transfer into the dialysate. The most common methods for estimating red blood parameters during HD are based on measurements of the hematocrit and hemoglobin concentration.

Studies^39,40^ showed that creatinine and phosphate clearances significantly decrease in the presence of high Hct values, although urea clearance is minimally affected and only demonstrates a negative trend. Urea, creatinine and phosphates are highly diffusible molecules and freely mobile between two different membrane surfaces.

Fleming et al. calculated the erythrocyte volume from mean corpuscular hemoglobin concentrations (MCHC), and found a significant correlation of RBC concentration and high dialysate sodium concentration^41^. Relative changes in RBC and Hb are strongly correlated (R = 0.96, p < 0.001) and lying relatively close one to another in our study. The difference between the Hb increase and Hct increase can be explained by the concomitant decrease in MCV of a similar percentage magnitude. If MCV remained unchanged during HD, the relative Hct variation would follow the changes in RBC. In such a case, the relative changes of Hct should follow those of Hb. The MCV decrease during HD was already reported by several authors in the past^42–44^. The study by Fleming et al.^41^ concluded that the dialysis fluid composition, mainly its sodium concentration, significantly influences plasma osmolarity and changes in MCV during dialysis. Also, during a typical dialysis session, MCV may transiently increase while the erythrocytes pass through the dialyzer^45^.

NIR data contains a huge amount of information, usually of very high dimension, which lends itself to the successful implementation of machine learning methods. Machine learning is a set of methods that can automatically detect patterns in data, and then use the detected patterns to make predictions on future data^46^. It is a relatively new but effective method that has been applied successfully in many fields.

The method used in this study is based on the surrogate indicators relating blood and dialysate constituents. The experimental results indicate a very good correlation between the NIR-absorbance spectrum of spent dialysate fluid and indicators of anemia and iron levels—RBC (0.93), Hb (0.92), Fe (0.94), TIBC (0.96), FER (0.91), Hct (0.94), MCV (0.92), MCH (0.93), MCHC (0.92) in the blood. The median value of the correlation coefficient for those solutes is high and the non-outlier range is very small when calculated over all 35 individual patients for whole spectrum ranging from 700–1700 nm.

More studies are needed to further evaluate the general validity of these results and elucidate the relationship between the parameters in dialyzate and blood. Further improvements in method precision might be expected with additional wavelength ranges, and by instrument improvements that will reduce or cancel noise.

The machine learning algorithms can be combined with other artificial intelligence methods or other statistical techniques, thus avoiding some of their limitations, such as the necessity of large amounts of data in order to successfully train a NN.

## Conclusions

In this work, a new approach combining ML and NIR scanning of the spent dialysis fluid has been proposed to enable fast, on-line evaluation of anemia in maintenance HD patients without the necessity of blood sampling. Neural networks have demonstrated a remarkable effectiveness in terms of efficiency (training time) and performance. It has been shown that the accuracy and precision of the proposed method for indirect determining the concentration of blood substituents can provide useful diagnostic screening information.

## Data Availability

Raw data were generated at Faculty of Mechanical Engineering, Belgrade University. Derived data supporting the findings of this study are available from the corresponding author on request.

## Code availability

Tools and functions from the MATLAB deep learning toolbox have been used in this study.
